# Music for pain relief during bed bathing of mechanically ventilated patients: A pilot study

**DOI:** 10.1371/journal.pone.0207174

**Published:** 2018-11-14

**Authors:** Gwenaëlle Jacq, Karine Melot, Mathilde Bezou, Laura Foucault, Josette Courau-Courtois, Sebastien Cavelot, Annie Lang, Jean-Pierre Bedos, Dominique Le-Boeuf, Jean-Marc Boussard, Stephane Legriel

**Affiliations:** 1 Intensive Care Department, GHT Sud Yvelines, Centre Hospitalier de Versailles—Site André Mignot, Le Chesnay Cedex, FRANCE; 2 Direction des soins, GHT Sud Yvelines, Centre hospitalier de Rambouillet, Rambouillet, FRANCE; University of Bern, SWITZERLAND

## Abstract

**Background:**

Pain is a universal issue and is of particular concern in mechanically ventilated patients, as they require intensive nursing care and multiple invasive procedures, while being unable to communicate verbally. The aim of this study was to assess the effect of music on pain experienced by mechanically ventilated patients during morning bed bathing.

**Methods:**

Of the 60 mechanically ventilated patients enrolled in this single-center pilot study between March 2013 and October 2015, the first 30 received no music and the next 30 the music intervention, during the morning bed bath. The Behavioral Pain Scale (BPS) score was determined during and at the end of the bath then 30, 60, and 120 minutes after the bath. BPS score changes over time were assessed and the proportions of bath times spent with a BPS score ≥5 and with the maximal BPS score were determined.

**Results:**

At baseline, no patient had pain (defined as a BPS score <5) and the median BPS score was 3 [IQR, 3;3] in both groups (*P* = 0.43). After bed bath initiation, 88% of patients experienced pain. The maximum BPS value during the bath was lower in the music group (5 [5;6.7] vs. 7 [5;7]). Proportions of total bath time spent with BPS≥5 and with the maximum BPS were significantly lower in the music group than in the control group (2.0 [0.3;4.0] vs. 10 [4.3;18.0]; *P* < .0001 and 1.5 [0;3.0] vs. 3.5 [2.0;6.0]; *P* = .005; respectively). Two hours after the end of the bath, the BPS values had returned to baseline in both groups.

**Conclusion:**

In our population, music significantly decreased pain intensity and duration during the morning bed bath in mechanically ventilated patients. These results warrant further assessment in a large multicenter randomized controlled trial.

**Trial registration:**

ClinicalTrials.gov NCT02883959

## Introduction

Among critically ill patients who are able to communicate, over 60% report pain [1, 2]. Pain is associated with the occurrence of delirium [3] and posttraumatic stress disorder [4]. The main sources of pain experienced by patients in the intensive care unit (ICU) are nursing care and invasive procedures (e.g., catheter care, drain removal, postoperative care, patient mobilization during bed bathing and other nursing care procedures, wound care, tracheal suctioning) [5, 6].

Pain control in ICU patients relies mainly on pharmacotherapy [7]. However, other methods warrant consideration [2]. Among them, music has been evaluated in many studies [8, 9]. The mechanisms underlying the effect of music in alleviating pain remain poorly understood. The main hypothesis is that processing music enhances endorphin release in the central nervous system, thereby elevating the pain threshold.[10] However, this effect has been demonstrated only in patients who actively practice music as opposed to only listen to music.[11] In critically ill patients receiving mechanical ventilation (MV), a Cochrane metaanalysis found that music decreased anxiety, heart rate, respiratory rate, and blood pressure.[12]. One possibility is that these beneficial effects were related to pain relief. To our knowledge, the effect of a music intervention on pain has not been specifically evaluated in the ICU.

The aim of this study was to assess the potential effect of a music intervention on pain experienced during morning bed bathing by patients receiving MV in the ICU. We conducted a prospective, comparative, single-center, nonrandomized pilot study to compare bed bathing with and without music.

## Materials and methods

### Ethics

Our local ethics committee approved this prospective pilot study (*Comité de Protection des Personnes*, *Paris–Ile de France VI*, 5 October 2012, #12044). When the first patient was enrolled on March 2013, which is 6 months after registration of the study protocol by the French health authorities, it was 3 years before clinicaltrials.gov registration (August 30, 2016). The authors confirm that not a single change was made in the study protocol during this time. The authors also confirm that all ongoing and related trials for this drug/intervention are registered. According to French law, written informed consent from a relative or surrogate was obtained for each participant before study inclusion. Written informed consent was then requested from all included patients as soon as they regained competence, and before ICU discharge. All procedures involving the patients complied with the ethical standards of the institutional and national research committees and with the 1964 Declaration of Helsinki and its later amendments.

### Patients

Adults admitted to the Versailles Hospital ICU between March 2013 and October 2015 were included prospectively if they were older than 18 years of age, were receiving MV, and had a Richmond Agitation Sedative Scale (RASS) (Table 1) [13] score between -3 and +4. Non-eligibility criteria for study inclusion were neuromuscular blocking agent therapy, being under guardianship, not being covered by the French statutory healthcare insurance system, having no relatives, being enrolled in another study, and being unwilling to participate. All patients who were ineligible for study inclusion were recorded prospectively in a register.

**Table 1:** Richmond agitation sedation scale (RASS score).

Score	Classification	(RASS)
**+4**	Combative	Overtly combative or violent; immediate danger to staff
**+3**	Very agitated	Pulls on or removes tube(s) or catheter(s) or has aggressive behavior toward staff
**+2**	Agitated	Frequent non-purposeful movement or patient–ventilator dyssynchrony
**+1**	Restless	Anxious or apprehensive but movements not aggressive or vigorous
**0**	Alert and calm	Spontaneously pays attention to caregiver
**-1**	Drowsy	Not fully alert, but has sustained (more than 10 seconds) awakening, with eye contact, to voice
**-2**	Light sedation	Briefly (less than 10 seconds) awakens with eye contact to voice
**-3**	Moderate sedation	Any movement (but no eye contact) to voice
**-4**	Deep sedation	No response to voice, but any movement to physical stimulation
**-5**	Unarousable	No response to voice or physical stimulation

### Participants

This single-center study was performed at the university-affiliated Versailles hospital ICU. The physicians and a clinical research nurse and/or assistant were in charge of daily patient screening and inclusion, ensuring compliance with the study protocol available in the data supplement as S1 and S2 Files, and collecting the study data in a case-report form. Some patients couldn’t be included simultaneously by the research team.

### Study design

We included 60 patients. The first 30 patients were included in the control group and the next 30 patients in the intervention group. As bed bathing is performed in all patients and in a standardized manner, we evaluated the potential effects of music during the morning bed bath. The Transparent Reporting of Evaluations with Nonrandomized Designs developed to guide standardized reporting of nonrandomized controlled trials is available in the data supplement as the S3 File.

### Standard care in both groups

Morning bed bathing was performed once a day at 8 AM by a nurse and nursing assistant. First, the anterior aspect of the body was washed with soap-based products. Ocular, nasal, and oral care was given. If needed, the patient was shaved and the endotracheal tube suctioned. Then, the patient was lateralized and the posterior aspect of the body and genitals were washed with soap-based products, a massage was given for pressure-sore prevention, and the sheets were changed.

All patients received analgesia and sedation as needed, according to the standard protocol in our unit, which involves various titrated combinations of midazolam, propofol, morphine, and/or sufentanil.

### Intervention

In the intervention group, patients were exposed to music during the morning bed bath, using headphones connected to an MP3 converter via a Bluetooth connection. The same selection of passages by Mozart was used in all patients[14, 15]. The music was started at the same time as the bed bath and continued throughout the bath and for 30 minutes after the end of the bath. Music amplitude was 60 decibels.

### Follow up and outcomes

The primary outcome was the proportion of total bath time spent with a BPS score ≥5, computed as the time spent with a BPS score ≥5 during the bath divided by the total bath time. The secondary outcome was the proportion of total bath time spent with the highest BPS score, computed as the time spent with the highest BPS score during the bath divided by the total bath time.

BPS scores (Table 2) [16] were determined by a nurse, who was not involved in providing the bath. The BPS score was determined just before the bath; at completion of the bath; and 30, 60, and 120 minutes after the end of the bath. During the bath, the BPS score was assessed as often as necessary when any of the score components differed from its baseline value. According to our protocol, for each patient, we identified the highest BPS score and the time spent with a BPS score ≥5 during the bed bath. Total length of the bath was recorded. All patients were followed-up to 120 minutes after the end of the bath.

**Table 2:** The Behavioral Pain Scale (BPS).

Item	Description	Score*
Facial expression	Relaxed	1
	Partially tightened (brow lowering)	2
	Fully tightened (eyelid closing)	3
	Grimacing	4
Upper limbs	No movement	1
	Partially bent	2
	Fully bent with finger flexion	3
	Permanently retracted	4
Compliance with ventilation	Tolerating movement	1
	Coughing but tolerating ventilation for most of the time	2
	Fighting ventilator	3
	Unable to control ventilation	4

*Total score can range from 3 (no pain) to 12 (maximum pain)

### Data collection

Demographic and clinical data were collected prospectively at the bedside on a standardized form. BPS scores were assessed as described above. Physiologic and respiratory parameters were collected automatically by a monitor (IntelliVue MX800, Phillips, Andover, MA) and central monitoring report system (IntelliVue IX, version A 01, Phillips). Sedation and analgesia, and physiologic parameters (systolic, diastolic, and mean arterial blood pressures, heart and respiratory rates, and pulse oxygen saturation) were assessed in both groups at baseline; during bed bathing; at the end of the bath, and 30, 60, and 120 minutes after the end of the bath.

Acute illness severity at ICU admission was assessed using the Simplified Acute Physiology Score II (SAPS-II),[17]. Length of ICU stay, time on MV, and vital status at ICU discharge were also collected.

### Statistical analysis

Quantitative parameters were described as median (interquartile range [IQR]) and qualitative parameters as number (%). Categorical variables were compared between treatment groups using Fisher’s exact test and continuous variables using the Wilcoxon test.

To test the robustness of our results, an exploratory analysis was carried out using a propensity score approach based on 1:1 pair matching when estimating associations between variables in the music intervention and control groups. We performed exact matching for each variable, with no replacement, using a specified caliper distance of 0.2. The variables included in the propensity score were selected among the demographic and baseline characteristics associated with treatment group by univariate statistical analysis (*P*<0.10). These variables were gender, age, SAPS II score, diastolic blood pressure, and number of invasive devices.

All tests were two-tailed at the 0.05 alpha level with a two-sided hypothesis. Analyses were performed using R statistical software version 3.3.1.3 (R Foundation for Statistical Computing, Vienna, Austria)*. *http://www.R-project.org. Accessed June 01, 2018

## Results

**Fig 1:**
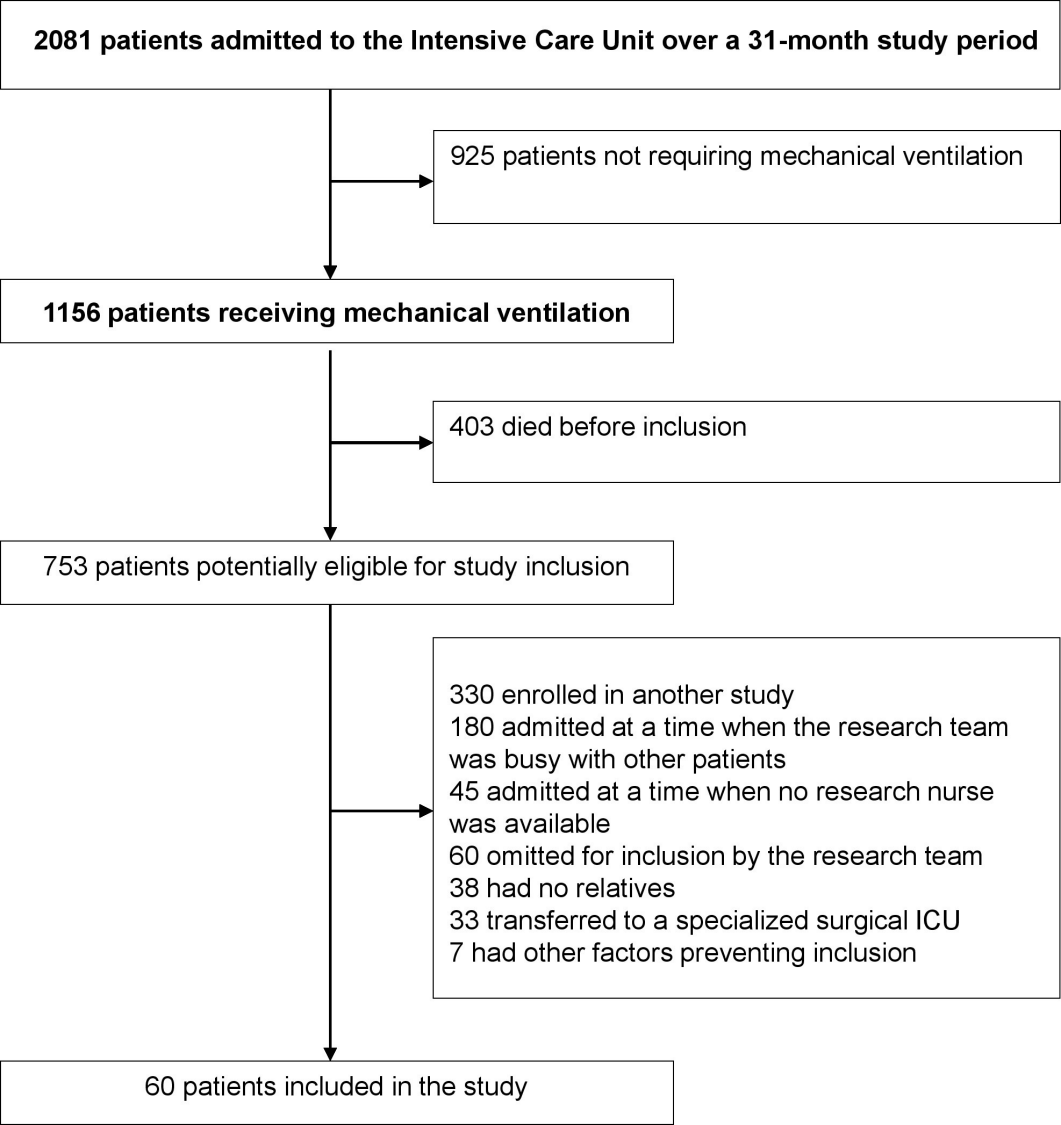
Study flow diagram.

Fig 1 is the study flow diagram.

### Patient characteristics

**Fig 2:**
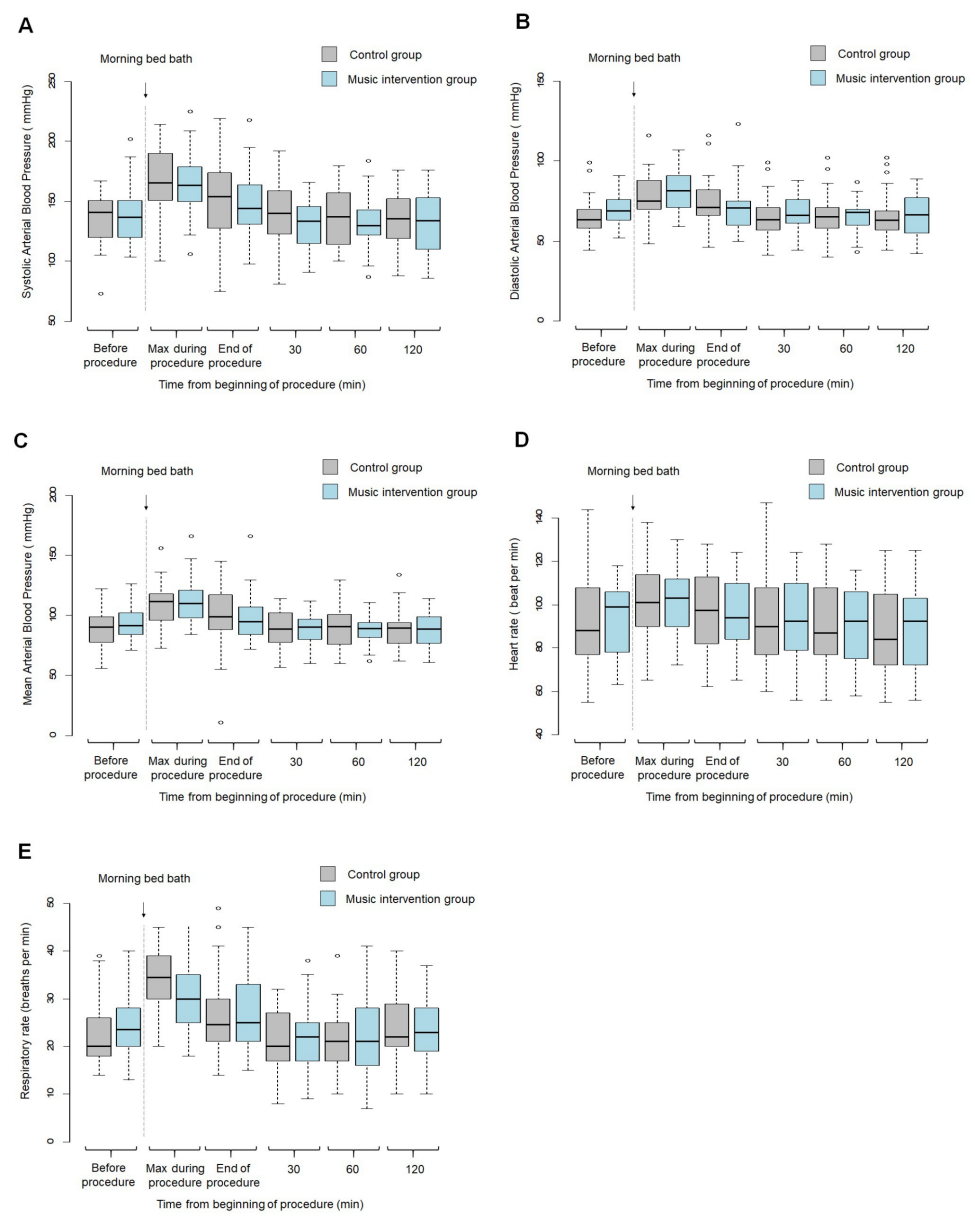
Changes in physiological parameters (systolic, diastolic, and mean arterial blood pressure and heart and respiratory rates) in each study group according to time from the beginning of the bed bath (n = 60). The shaded area indicates the middle 50% of the data and the lower and upper ends of the box the 25th percentile and 75th percentile, respectively. The solid black horizontal line through each shaded box indicates the median value. The circles above the vertical solid black lines are individual outliers. *P* values are provided above each pair of values. Gray color indicates the control group and blue color the music intervention group. Panel A. Boxplot of systolic arterial blood pressure (mmHg) in the music and control groups. Panel B. Boxplot of diastolic arterial blood pressure (mmHg) in the music and control groups. Panel C. Boxplot of mean arterial blood pressure (mmHg) in the music and control groups. Panel D. Boxplot of heart rate (beat per min) in the music and control groups. Panel E. Boxplot of respiratory rate (beat per min) in the music and control groups.

Table 3 reports the main patient characteristics in each group. Median age of the overall population was 69 [60;80] years. Most patients were admitted for medical reasons and had respiratory or hemodynamic failure as the reason for MV. The median SAPS II values indicated severe illness. Only a minority of patients were receiving sedatives and/or analgesics before the bed bath. Fig 2A–2E report the changes in physiological parameters (systolic, diastolic, and mean arterial blood pressures and heart and respiratory rates) in each group at each time point from the start of the bath.

**Table 3:** Patient characteristics at inclusion (n = 60).

	Music intervention	Control	
	N = 30	N = 30	*P* value
**Demographics**			
Age, years, median [IQR]	78 [63;80]	65 [59;77]	.09
Male gender, n (%)	11 (36.7)	20 (66.7)	.04
SAPS II, median [IQR]	49.5 [44;63]	62 [49;72]	.06
Reason for ICU admission, n (%)			
Medical/Emergent surgery versus Scheduled surgery	28 (93.3)	29 (96.7%)	1.00
Number of comorbidities median [IQR]	2 [1;2]	2 [2;3]	1.00
**Characteristics at inclusion**			
RASS score, median [IQR]	0 [-0.7;0]	0 [-1;0]	.19
Reason for mechanical ventilation, n (%)			.70
Respiratory failure	11 (36.7)	11 (36.7)	
Hemodynamic failure	12 (40.0)	11 (36.7)	
Post-surgical care	4 (13.3)	2 (6.6)	
Neurological failure	3 (10.0)	6 (20.0)	
Ventilator mode, n (%)			1.00
A/C	3 (10.0)	2 (6.7)	
SPN-PPS	27 (90.0)	28 (93.3)	
MV duration at inclusion, days, median [IQR]	5 [3;13]	5 [3;12]	.94
SpO_2_ (%) median [IQR]	97 [95; 98]	96 [95;98]	.58
Respiratory rate, breaths/min, median [IQR]	24 [20;28]	20 [18;26]	.14
Systolic blood pressure, mmHg, median [IQR]	137 [121;151]	141 [122;150]	.90
Diastolic blood pressure, mmHg, median [IQR]	69 [64;75]	64 [58;69]	.05
Mean blood pressure, mmHg, median [IQR]	92 [85;101]	91 [78;99]	.38
Heart rate, beats/min, median [IQR]	99 [79;105]	88 [77;108]	.44
Patients receiving vasoactive drugs, n (%)	3 (10.0)	6 (20.0)	.47
Patients receiving sedatives, n (%)	7 (23.0)	10 (33.3)	.57
Propofol	6	9	-
Midazolam	1	0	-
Other	0	1	-
Patients receiving analgesics, n (%)	5 (16.7)	9 (30.0)	.36
Sufentanil	5	7	-
Morphine	0	2	-
Number of invasive devices, median [IQR]	4 [4;5]	4 [3;4]	.02

Abbreviations: IQR, interquartile range; ICU, intensive care unit; SAPS II, Simplified Acute Physiology Score version II; RASS, Richmond Agitation-Sedation Scale; A/C, assist-control ventilation; SPN-PPS, spontaneous proportional pressure support; SpO_2_, pulse oximeter oxygen saturation

### Behavioral Pain Scale (BPS) values

**Fig 3:**
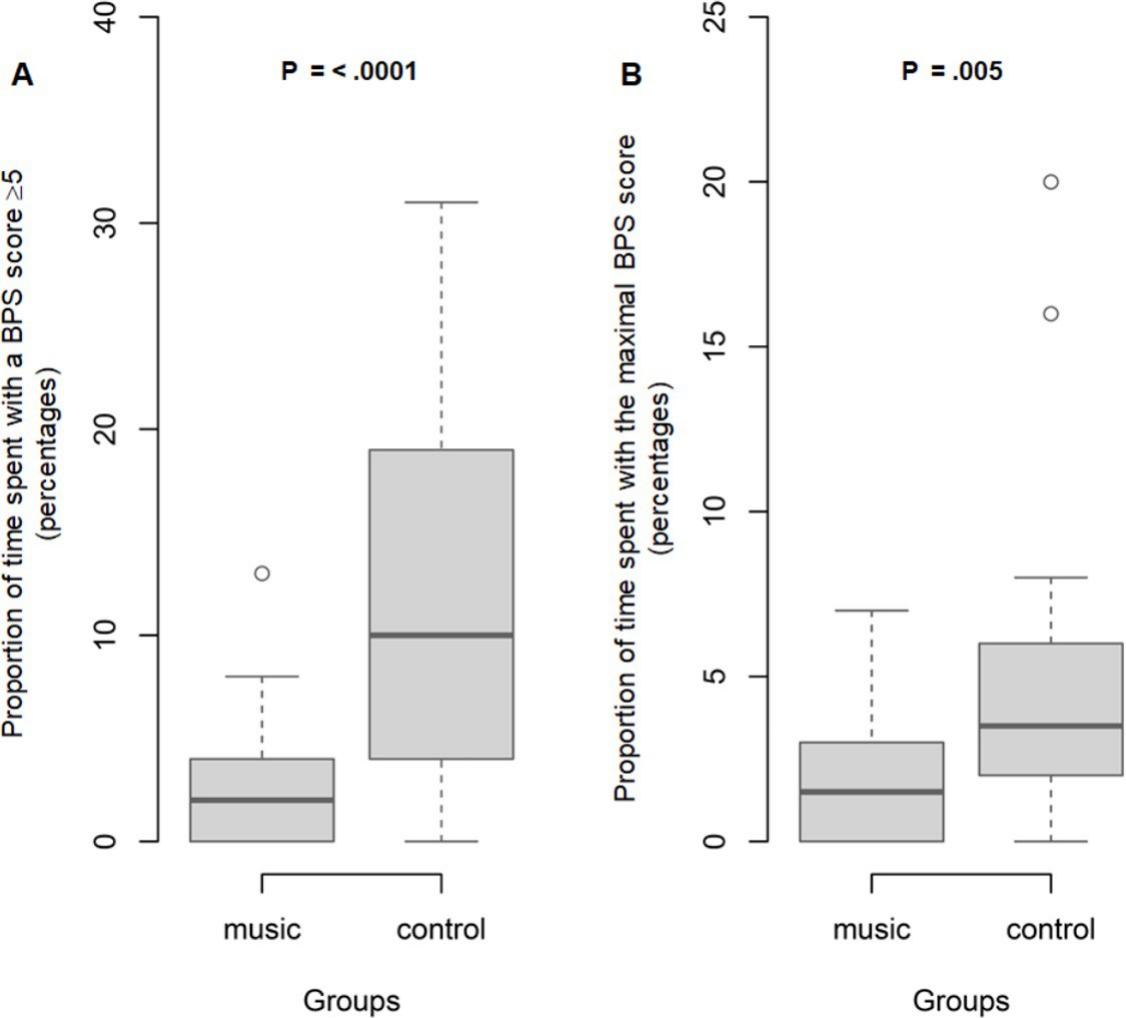
**Boxplot of proportion of total bath time spent with Behavioral Pain Scale (BPS) score ≥5 (Panel A) and with maximal BPS score (Panel B) in the music and control groups (n = 60).** The shaded area indicates the middle 50% of the data and the lower and upper ends of the box the 25th percentile and the 75th percentile, respectively. The solid black horizontal line through each shaded box indicates the median value. The circles above the vertical solid black lines are individual outliers. *P* values are provided above each pair of values. Panel A. Boxplot of total time spent with Behavioral Pain Scale (BPS) score ≥5 in the music and control groups. Panel B. Boxplot of proportion of total bath time spent with maximal Behavioral Pain Scale (BPS) score in the music and control groups.

Table 4 reports the BPS scores in the music intervention and control groups. At baseline, no patient had pain and the median BPS score was 3 [IQR, 3;3] in both groups (*P* = .43). After bed bath initiation, 88% of patients experienced pain (as defined by a BPS score ≥5). The maximum BPS value during bathing was lower in the music group (5 [5;6.7] vs. 7 [5;7]). Fig 3A and 3B reports the proportions of time spent with BPS≥5 and with the maximum BPS. Proportions of total bath time spent with a BPS score ≥5 and with the maximum BPS score were significantly lower in the music group than in the control group (2.0 [0.3;4.0] vs. 10 [4.3;18.0]; *P* < .0001) and (1.5 [0;3.0] vs 3.5 [2.0;6.0]; *P* = .005); respectively. Two hours after the end of the bath, the BPS values had returned to baseline in both groups.

**Table 4:** Behavioral Pain Scale (BPS) scores in the music intervention and control groups.

	Groups	
	Median (Interquartile Range)	
	Music intervention	Control
	n = 30	n = 30
BPS (just before the bed bath)	3 [3;3]	3 [3;3]
BPS (during the bed bath)		
Minimum	3 [3;3]	3 [3;3]
Maximum	5 [5;7]	7 [5;7]
BPS (Post procedure)		
End of procedure	3 [3;3]	3 [3;4]
30 min after the end of procedure	3 [3;3]	3 [3;3]
60 min after the end of procedure	3 [3;3]	3 [3;3]
120 min after the end of procedure	3 [3;3]	3 [3;3]
Total time spent with pain (seconds)*	31 [7;57]	122 [55;227]
Total time spent with maximal pain (seconds)*	18 [6;37]	44 [17;59]
Total bed bath duration (seconds)	1320[1080;1560]	1230[1050;1380]

*As evaluated by the BPS score.

### Sensitivity analysis

**Fig 4:**
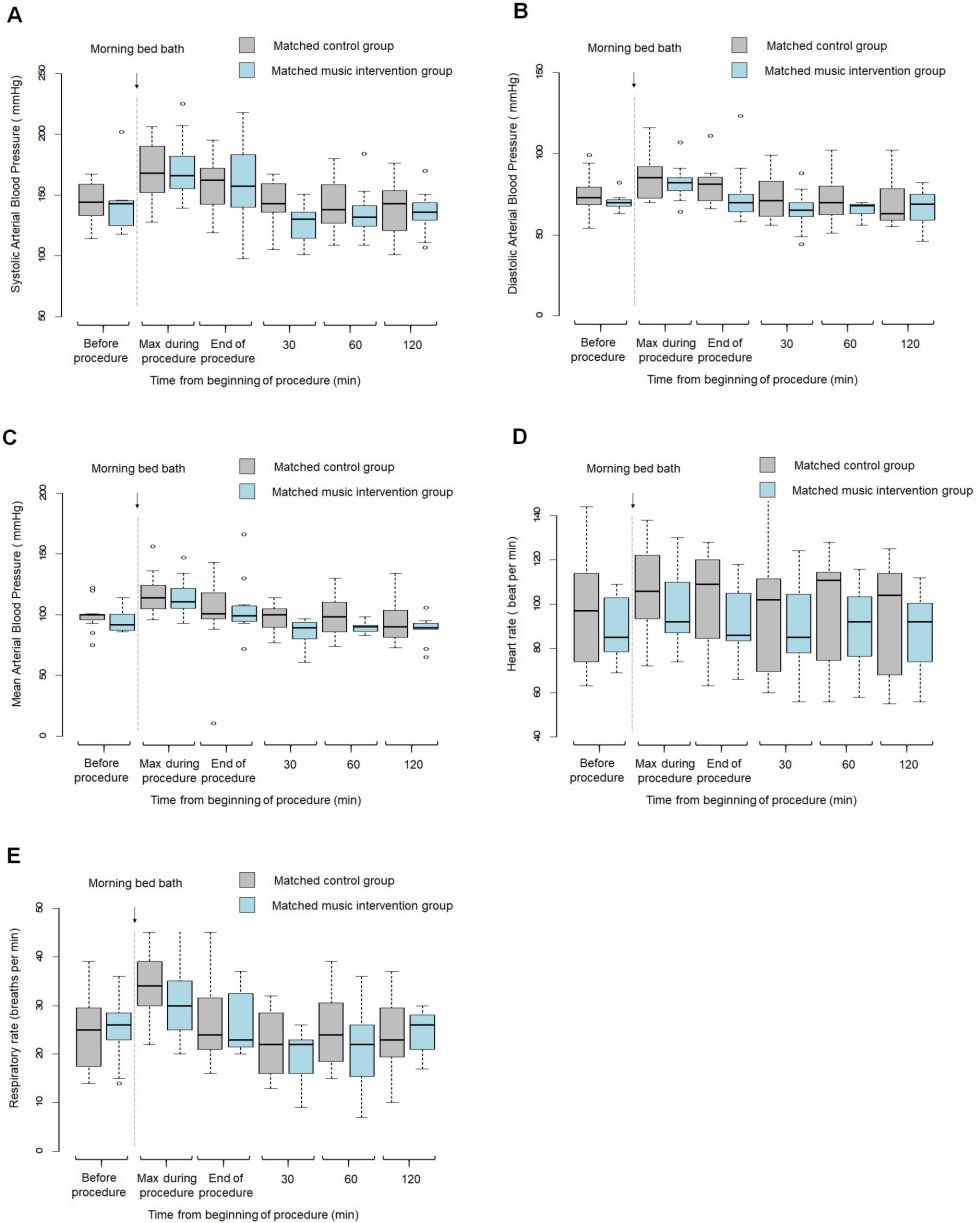
Analysis in the matched subsample: Changes in physiological parameters (systolic, diastolic, and mean arterial blood pressure and heart and respiratory rates) in each study group according to time from the beginning of the bed bath (n = 22). The shaded area indicates the middle 50% of the data and the lower and upper ends of the box the 25th percentile and the 75th percentile, respectively. The solid black horizontal line through each shaded box indicates the median value. The circles above the vertical solid black lines are individual outliers. *P* values are provided above each pair of values. Gray color indicates the matched control group and blue color the matched music intervention group. Panel A. Boxplot of systolic arterial blood pressure (mmHg) in the matched music and control groups. Panel B. Boxplot of diastolic arterial blood pressure (mmHg) in the matched music and control groups. Panel C. Boxplot of mean arterial blood pressure (mmHg) in the matched music and control groups. Panel D. Boxplot of heart rate (beats per min) in the matched music and control groups. Panel E. Boxplot of respiratory rate (beat per min) in the matched music and control groups.

**Fig 5:**
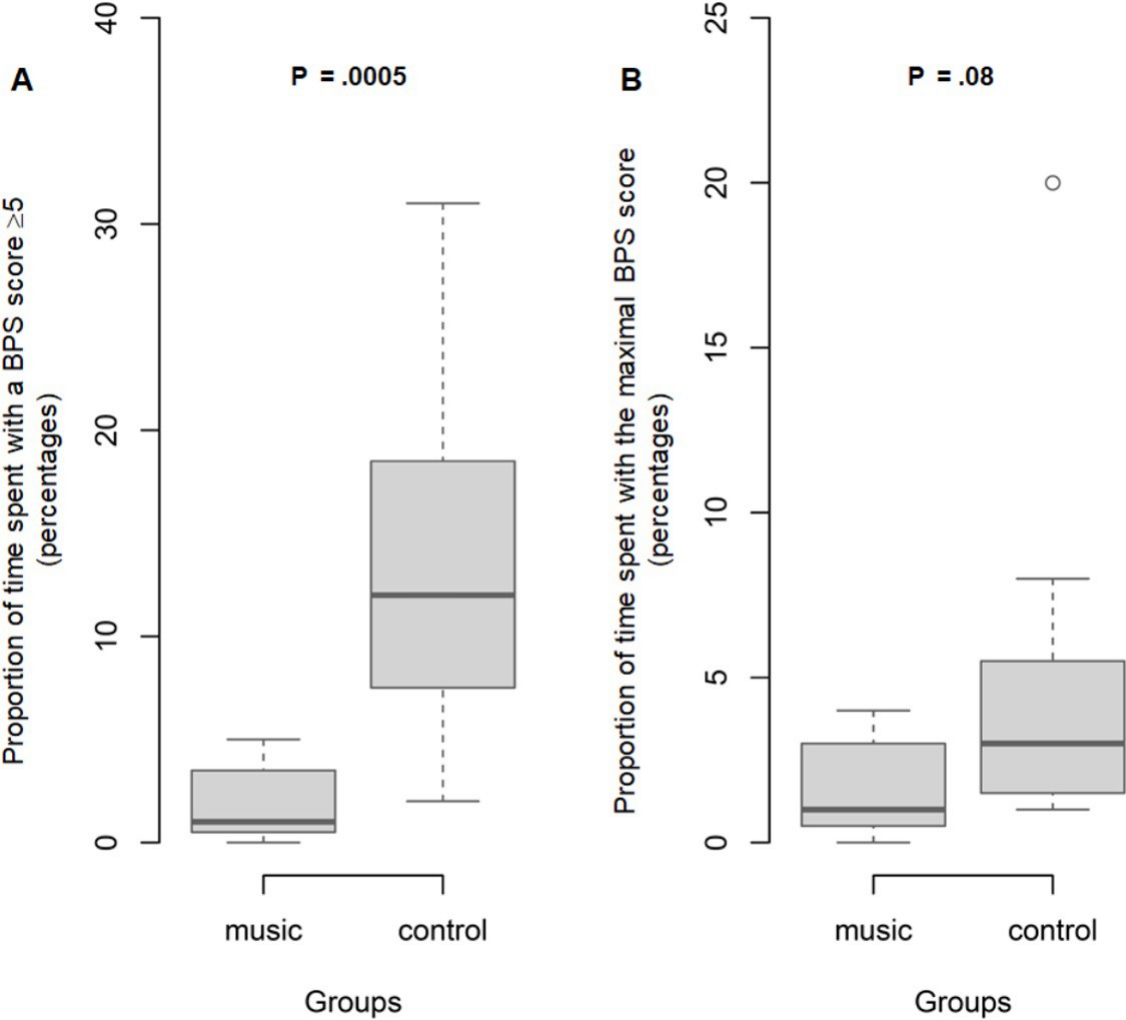
**Boxplot of the proportion of total bath time spent with Behavioral Pain Scale (BPS) score ≥5 (Panel A) and with the maximal Behavioral Pain Scale (BPS) score (Panel B) in the matched music and control groups (n = 22)**. The shaded area indicates the middle 50% of the data and the lower and upper ends of the box the 25th percentile and the 75th percentile, respectively. The solid black horizontal line through each shaded box indicates the median value. The circles above the vertical solid black lines are individual outliers. *P* values are provided above each pair of values. Panel A. Boxplot of total time spent with Behavioral Pain Scale (BPS) score ≥5 in the matched music and control groups. Panel B. Boxplot of proportion of total bath time spent with the maximum Behavioral Pain Scale (BPS) score in the matched music and control groups.

Tables 5 and 6 report the main patient characteristics and BPS scores in 11 patients from each group matched in pairs on a propensity score. Fig 4A–4E reports the changes in physiological parameters (systolic, diastolic, and mean arterial blood pressures and heart and respiratory rates) at each time point from the start of the bath in the matched music and control groups. As illustrated in Fig 5A and 5B, the results in the matched subsample were comparable to those in the overall population: in the music group, the maximum BPS value during the bath was lower and the total times spent with a BPS score ≥5 and with the maximal BPS score were shorter. The proportion of total bath time spent with BPS≥5 remained significantly shorter in the music group than in the control group (1.0 [0.5;3.5] vs. 12 [7.5;18.5]; *P* = .0005), the proportion spent with the maximum BPS was not significantly different between the two groups (1.0 [0.5;3.0] vs. 3.0 [1.5;9.5]; *P* = .08).

**Table 5:** Patient characteristics in 11 patients from the music intervention and control groups who were pair-matched on a propensity score.

	Music intervention	Control	
	n = 11	n = 11	*P* value
**Demographics**			
Age, years, median [IQR]	65 [62;80]	65 [60;79]	1.00
Male gender, n (%)	6 (55.5)	6 (55.5)	1.00
SAPS II, median [IQR]	62 [57;69]	63 [51;75.5]	1.00
Reason for ICU admission, n (%)			
Medical/Emergent surgery versus Scheduled surgery	11 (100)	11 (100)	1.00
Number of comorbidities median [IQR]	2 [0.5;2]	2 [1.5;3]	0.47
**Characteristics at inclusion**			
RASS score, median [IQR]	0 [0;0]	0 [-1;0]	0.33
Reason for mechanical ventilation, n (%)			0.45
Respiratory failure	6 (54.5)	4 (36.7)	
Hemodynamic failure	3 (27.3)	3 (27.3)	
Post-surgical care	0	0	
Neurological failure	1 (9.1)	4 (36.7)	
Ventilator mode, n (%)			1.00
A/C	0	0	
SPN-PPS	11 (100)	1 (9.1)	
MV duration at inclusion, days, median [IQR]	11 [7;15]	5 [4;10]	0.10
SpO_2_ (%) median [IQR]	96 [95;97]	96 [96;98]	0.61
Respiratory rate, breaths/min, median [IQR]	26 [23;29]	25 [18;30]	0.64
Systolic blood pressure, mmHg, median [IQR]	143 [125;146]	144 [133;159]	0.36
Diastolic blood pressure, mmHg, median [IQR]	70 [68;72]	73 [69;79]	-
Mean blood pressure, mmHg, median [IQR]	92 [88;101]	100 [96;101]	0.32
Heart rate, beats/min, median [IQR]	85 [79;103]	97 [94;114]	0.69
Patients receiving vasoactive drugs, n (%)	0	1 (10.1)	-
Patients receiving sedatives, n (%)	2 (18.2%)	2 (18.2%)	1.00
Propofol	2	2	-
Patients receiving analgesics, n (%)	2 (18.2%)	2 (18.2%)	1.00
Sufentanil	2	1	-
Morphine	0	1	-
Number of invasive devices, median [IQR]	4 [3;4]	4 [3;4]	1.00

Abbreviations: IQR, interquartile range; ICU, intensive care unit; SAPS II, Simplified Acute Physiology Score version II; RASS, Richmond Agitation-Sedation Scale; A/C, assist-control ventilation; SPN-PPS, spontaneous proportional pressure support; SpO_2_, pulse oximeter oxygen saturation

**Table 6:** Behavioral Pain Scale (BPS) scores in 11 patients from the music intervention and control groups who were pair-matched on a propensity score.

	Groups	
	Median (Interquartile Range)	
	Music intervention	Control
	n = 11	n = 11
BPS (just before the bed bath)	3 [3;3]	3 [3;3]
BPS (during the bed bath)		
Minimum	3 [3;3]	3 [3;3]
Maximum	6 [5;7]	7 [6;8]
BPS (Post procedure)		
End of procedure	3 [3;3]	4 [3;4]
30 min after the end of procedure	3 [3;3]	3 [3;3]
60 min after the end of procedure	3 [3;3]	3 [3;3]
120 min after the end of procedure	3 [3;3]	3 [3;3]
Total time spent with pain (seconds)*	23 [6;41]	142 [90;258]
Total time spent with maximal pain (seconds)*	14 [4;41]	45 [15;54]
Total bed bath duration (seconds)	1260 [1080;1380]	1140 [900;1350]

*As evaluated by the BPS score.

## Discussion

To our knowledge, this study provides the first information on the effects of a music intervention on pain during a potentially painful nursing care procedure in critically ill patients receiving mechanical ventilation (MV). Of the 60 prospectively included patients, 88% had pain, defined as a Behavioral Pain Scale (BPS) score ≥5, during bed bathing. Total times spent with a BPS score ≥5 and with the maximal BPS score were significantly shorter in the music intervention group compared to the controls.

Pain control in ICU patients is a major but challenging treatment objective that is receiving increasing attention [2]. Pain intensity and duration vary widely depending on patient factors, the underlying disease, the critical illness, and the treatment and nursing interventions that must be provided. Nevertheless, some procedures are universally painful, such as chest tube removal, wound drain removal, and arterial line insertion [18]. Bed bathing is not only necessary to maintain hygiene, but also potentially a relaxing procedure for the patient [19]. In ICU patients, however, bed bathing may induce pain due to the presence of invasive material (endotracheal tube, catheters, drains) and need to mobilize the patient. In a large multinational study, turning, positioning, and mobilization were shown to induce pain [1] [18]. Moreover, the morning bed bath is often combined with other potentially painful procedures such as tracheal suctioning[20].

Our findings are consistent with previous studies of pain during nursing care in the ICU. In keeping with these data, 88% of our patients experienced pain during bed bathing. The heterogeneity of previously published studies of pain in ICU patients hinders comparisons. Among unselected critically ill patients, including 37% on MV and 65 able to communicate, 63% experienced pain and 15% severe pain during the ICU stay [1]. When questioned after ICU admission for post-cardiac surgery care, 77% of patients recalled experiencing pain during the ICU stay [21]. In a population of unselected ICU patients receiving MV, i.e., similar to our population, 56% of patients experienced pain when evaluated during nursing care procedures [22]. This lower proportion than in our study may be related to the use of sedatives and/or analgesics in 90% of patients, compared to only 27% of our patients. Other potential sources of variation across studies include differences in the care procedures evaluated, pain assessment modalities, and cutoffs used to define pain.

We believe our findings provide a new contribution that may have clinical and scientific implications. The authors of a 2014 Cochrane review[12] concluded that music interventions alleviated anxiety in critically ill patients receiving MV. However, they did not specifically assess the effect of music interventions on pain, although they did report that music interventions were associated with lower sedative and analgesic intakes. The original feature of our study is that we focused specifically on pain, as assessed using the validated BPS. In our study, music was associated with a powerful analgesic effect manifesting as significant decreases in times spent with a BPS score ≥5 and with the maximal BPS score during morning bed bathing. The potential analgesic effects of music have been evaluated in several previous studies in the fields of pediatrics[23], oncology[24], surgery[25], and chronic pain[26]. A Cochrane metaanalysis of studies in patients receiving MV in the ICU showed that music decreased anxiety, heart rate, respiratory rate, and blood pressure.[12] These physiological changes suggest that music may act on the sensory, cognitive, affective, and behavioral components of pain defined by the International Association for the Study of Pain. However, few studies specifically addressed the ability of music to lessen pain in ICU patients receiving MV.[27–29] Music was associated with a significant decrease in pain during and up to 20 minutes after tracheal suctioning, although no differences with the control group occurred for blood pressure, heart rate, or oxygen saturation.[30] In contrast, we observed changes in all the physiological parameters measured for the study between the start and end of the bed bath. All these parameters were altered during bathing then improved gradually, returning to baseline within 2 hours after the end of the bath. Interestingly, the physiological parameters showed trends toward better values in the music intervention group, both in the overall population and in the matched subsample. These apparent discrepancies with earlier studies may be related to the difference in the methods used to assess the effects of music. Bed bathing lasts longer than does tracheal suctioning and involves multiple potentially painful events, which may include tracheal suctioning. Finally, although we focused on pain intensity, consumption of sedatives and/or analgesics differed between the two groups [31–33]. Nevertheless, our matched subsample analysis produced two groups with similar sedative and analgesic intakes yet also showed less pain in the music group.

Our study has several limitations. The single-center design mandates caution regarding the applicability of our findings to the full spectrum of nursing care procedures. Moreover, several potential sources of biases must be acknowledged. First, for this pilot study, randomization was not used, and bias in patient selection may therefore have occurred. Thus, the two groups differed significantly regarding gender distribution, diastolic blood pressure, and number of invasive devices. To minimize the consequences of potential patient selection bias, we performed a sensitivity analysis after matching patients in the music intervention and control groups on a propensity score based on the demographic and baseline characteristics associated with treatment group by univariate statistical analysis (*P*<0.10). Second, the nurse who conducted the BPS score assessments was not blinded to the treatment group, potentially leading to confirmatory bias. Third, the music was selected by the researchers and may not have matched patient preferences. Fourth, although our endpoint of pain was assessed by having a specifically trained and experienced nurse apply a well-validated and reproducible pain scale, the BPS, neuromonitoring was not used as a tool for comparing pain in the two groups.[34–36] Additionally, nurse expertise or quality of standard care may have changed between the initial data collection for control patients and the subsequent data collection for patients receiving the music intervention. However, in both groups, we collected data on objective physiological parameters including systolic, diastolic, and mean blood pressures, as well as heart and respiratory rates, starting just before and continuing until the end of the assessment period. Trends toward decreases in these parameters were found in the intervention group compared to the control group. Thus, despite the absence of randomization and non-blinded assessment of the endpoint, we believe our study does provide valid data on pain during a bed bath in mechanically ventilated ICU patients who are unable to communicate verbally. Fifth, we did not evaluate the patients’ perceptions of the music they were exposed to. Thus, we cannot exclude that listening to Mozart may have been unpleasant for some patients. However, we used the same music in all patients to standardize our intervention. Finally, our choice was guided by current knowledge indicating a positive effect of classical music in critically ill patients.[15] Despite these limitations, our pilot study provides information that should prove useful for designing a large randomized trial.

## Conclusion

In conclusion, in our population, a simple music intervention was associated with significant decreases in pain intensity and duration during bed bathing in patients receiving MV in the ICU. These results warrant further evaluation in a large multicenter randomized controlled trial.

## Supporting information

S1 File Protocol French version. (PDF)

S2 File Protocol English version. (PDF)

S3 File Trend statement checklist. (PDF)

## Abbreviations

BPS: Behavioral Pain Scale
RASS: Richmond Agitation Sedation Scale
ICU: intensive care unit
IQR: interquartile range
