# Supplementary material for: Music for pain relief during bed bathing of mechanically ventilated patients: A pilot study
Source: PLoS One. 2018 Nov 14;13(11):e0207174. doi: 10.1371/journal.pone.0207174 (PMC6235356; doi:10.1371/journal.pone.0207174)
Supplement: S1 File — (PDF) [file pone.0207174.s001.pdf]

Evaluation de l'effet antalgique de la musicothérapie chez le patient de réanimation non communicant lors de procédures de soins infirmiers potentiellement douloureuses : Etude pilote

Etude PainKiller 1

Etat des connaissances

La douleur est une problématique fréquente s'opposant au mieux vivre en réanimation, rapportée chez plus de 60% des patients communicants.[1, 2] Elle est associée à une morbidité liée à la survenue de delirium[3] et de stress post-traumatique.[4] En réanimation, ses causes sont nombreuses et essentiellement liées aux soins et actes invasifs (pose de cathéters, drains, post-opératoire, mobilisations du patient lors de la toilette et soins de nursing, pansements divers, aspirations trachéales…).[5, 6] Sa prise en compte repose sur une stratégie simple qui associe : prévention, évaluation, et traitement médicamenteux et non médicamenteux de la douleur. Des recommandations récentes ont été formulées dans ce sens.[7]

L'évaluation de la douleur reste cependant une problématique difficile et souvent sous estimée en réanimation.[6] L'utilisation d'outils d'évaluation de la douleur est indispensable et permet une meilleure adaptation de l'analgésie par les équipes soignantes.[8] Chez le patient communiquant, l'indicateur le plus fiable est l'autoévaluation à l'aide des échelles EVA-ENS-EN.[7] Chez le patient non communicant, il est recommandé d'utiliser l'échelle d'hétéro évaluation comportementale de douleur BPS (Behavioral Pain Scale) qui a été évaluée comme fiable et reproductible.[9]

Alors que la prise en charge de la douleur en réanimation repose essentiellement sur la prescription de traitements médicamenteux [6], plusieurs études ont récemment montré l'intérêt de stratégies de sédation séquentielles (diminution de la durée de ventilation mécanique, diminution de la durée de séjour en réanimation, diminution de l'incidence du delirium)[10, 11], exposant les patients à un risque algique accru en cas de négligence de cet aspect de la prise en charge. Pour certains, cette stratégie est associée à une recrudescence de la morbidité, liée à des conséquences non seulement à court terme (désadaptation du ventilateur, agitation, tachycardie, hypertension, autoextubations…)[12] mais également à long terme (syndrome de stress post traumatique)[13]

Il apparaît donc important de pouvoir proposer des stratégies de prise en charge complémentaires de la douleur. La musicothérapie est l'une d'elles et a pu être évaluée dans de nombreux travaux scientifiques.[14] Elle a particulièrement été étudiée dans certains domaines d'application tels que la pédiatrie[15-18], la cancérologie[19-21], ou la neuro-psychiatrie.[22-24] L'évaluation de l'intérêt analgésique de la musicothérapie a fait récemment l'objet d'une méta-analyse du groupe Cochrane qui conclue à un bénéfice en termes de diminution de l'intensité de la douleur et du recours aux analgésiques médicamenteux.[14] Dans le domaine de la réanimation, chez les patients placés sous ventilation mécanique, les effets de la musicothérapie ont été évalués par une autre méta-analyse du groupe Cochrane montrant une diminution de l'anxiété, du rythme cardiaque, de la fréquence respiratoire mais aussi de la pression artérielle.[19, 25] Cette approche est intéressante et repose sur des mécanismes physiologiques maintenant mieux connus. L'action de la musicothérapie a ainsi été démontrée sur les différentes composantes de la douleur (Figure 1):

- sur la composante **sensorielle** (activation des voies de la douleur et de la transmission cérébrale avec modulation et contrôle des influx douloureux aux différents étages du système nerveux) : en exerçant un effet inhibiteur sur les fibres afférentes.[26]

- sur la composante **cognitive** (représentation psychique de la douleur par rapport aux expériences douloureuses antérieures et aux événements associés aux épisodes douloureux) **:** en détournant l'attention par des souvenirs ou images.[27]

- sur la composante **affective** (confère à la douleur un caractère plus ou moins pénible, désagréable, supportable selon les individus, peut même évoluer vers des états d'anxiété ou de dépression) **:** en modifiant l'état d'humeur et en stimulant la libération d'endorphines.[28]

- sur la composante **comportementale** (comportement de l'individu en cas de situation douloureuse) **:** en agissant sur l'hypertonie et la psychomotricité.

Ainsi, à l'heure où les recommandations d'experts incitent à élaborer des outils d'évaluation et à développer des comportements et des organisations soignantes qui permettent aux patients de « mieux vivre la réanimation »[2], il parait intéressant d'évaluer la valeur thérapeutique de la musique comme intervention en soins infirmiers lors des gestes potentiellement douloureux en réanimation afin d'améliorer la prise en charge des patients.

Buts de l'évaluation

Objectif principal

Evaluer le temps d'exposition à la douleur chez les patients de réanimation non communicants, placés sous ventilation mécanique, recevant ou non de la musicothérapie, lors de soins infirmiers.

Objectif secondaire

Evaluer la survenue d'une douleur paroxystique (pic de douleur) chez les patients de réanimation non communicants, placés sous ventilation mécanique, recevant ou non de la musicothérapie, lors de soins infirmiers.

Critère de jugement principal

La douleur sera évaluée par le temps d'exposition (en minutes) à une valeur de l'échelle BPS ≥ 5, rapporté au temps total d'exposition à l'acte potentiellement douloureux. Elle sera exprimée en pourcentage de temps de douleur au cours de l'acte infirmier étudié.

Critères de jugement secondaire

La douleur sera évaluée par la valeur maximale de douleur mesurée par l'échelle BPS lors de l'exposition à l'acte infirmier étudié. Elle sera exprimée en valeur d'échelle BPS.

Type d’évaluation

Etude prospective, mono-centrique, interventionnelle

Nombre de sujets nécessaires et durée de l’évaluation

Il s’agit d’une étude pilote dont le but est d’obtenir des données préliminaires et pour laquelle il n’est pas possible de calculer un nombre de sujets nécessaires. Le nombre de patients à inclure a été fixé à 60, soit 15 patients dans chaque groupe (toilette et pansements chirurgicaux, avec et sans musicothérapie).

Méthode

Critères d’inclusion

- patients adultes (âge≥18 ans) hospitalisés en Réanimation
- placés sous ventilation mécanique invasive, et non communicants verbalement
- dont le score de sédation, évalué par l’échelle RASS, est compris entre [-3 ; +4]

Critères de non inclusion

- patients curarisés
- patients dont score de sédation, évalué par l’échelle RASS est < -3

<u>Protocole d'étude</u>

Modalités de recrutement des patients

Cette évaluation vise à inclure consécutivement tous les patients éligibles. Les trente premiers patients éligibles seront inclus dans le groupe standard où aucune séance de musicothérapie ne sera appliquée ; les trente patients suivant seront inclus dans le groupe « musicothérapie » où la procédure de soin se déroulera associée à une séance de musicothérapie.

Soins évalués

Dans les 2 groupes un seul acte sera évalué par patient. Les actes infirmiers qui feront l'objet de l'évaluation sont des actes réalisés de manière routinière, réalisant une évaluation de soins courant. Les actes suivants pourront être évalués : toilette et pansements chirurgicaux.

Modalités pratiques de réalisation de la musicothérapie

La séance de musicothérapie débutera au début du soin et se poursuivra jusqu'à 30 minutes après la fin du soin. Le patient sera isolé de l'environnement sonore à l'aide d'un casque audio permettant la diffusion de la musique, dont l'intensité du niveau sonore ne devra dépassera pas 60 décibels. La musique diffusée sera standardisée et identique pour tous les patients (sélection standardisée « apaisante » de musique classique du compositeur Mozart). L'ensemble des évaluations réalisées lors du soin seront identiques à celles du bras standard. Le relevé des données à 30 minutes de la fin du soin sera réalisé avant l'arrêt de la musique. Les relevés suivants sont réalisés sans musique.

<u>Modalités communes aux 2 bras d'évaluation</u>

Dans les 2 bras d'évaluation, le soin infirmier évalué sera réalisé de manière habituelle.

Afin de ne pas perturber la prise en charge du patient, et permettre une évaluation fiable et standardisée, l'évaluation de la douleur et le relevé des données seront réalisés par un(e) infirmier(e) DE ne participant pas directement au soin.

L'évaluateur relèvera les différents horaires de réalisation du soin, et quantifiera le temps d'exposition à une douleur correspondant à une valeur de l'échelle BPS ≥ 5, ainsi que le score BPS maximum observé au cours du soin et ce jusqu'à 2 heures après la fin de ce dernier.

En cas de douleur, les patients bénéficieront d'une prise en charge habituelle reposant sur l'administration d'antalgiques selon une stratégie d'administration graduée conforme aux recommandations formulées dans les recommandations « Sédation-analgésie en réanimation » et dont le choix de la molécule utilisée, sa posologie et le mode d'administration sont laissés à la discrétion du médecin prenant en charge le patient.

Données relevées

Dans les 2 bras d'évaluation, on relèvera à l'aide d'un formulaire standardisé :

- données démographiques: âge, sexe, type d'admission (médical, chirurgical urgent ou programmé), les comorbidités associées, le motif de mise sous ventilation mécanique, la durée de séjour avant la réalisation du soin.

- données liées à l'environnement direct du patient : la présence d'une trachéotomie, de drains thoraciques, sonde gastrique, voie veineuse centrale, cathéter artériel, système de drainage chirurgical.

- données liées au soin infirmier (avant, et à 30 minutes, 1 heure et 2 heures après la fin du soin): type de soin évalué, durée totale du soin, paramètres hémodynamiques et ventilatoires, score RASS, administration concomitante de traitements sédatifs et/ou analgésiques.

- données liées à l'évaluation de la douleur : score BPS maximal et durée totale d'exposition à un score BPS ≥ 5.

Aspects législatifs et éthiques

Les soins infirmiers réalisés correspondent à des soins couramment réalisés chez les patients de réanimation. Aucune intervention supplémentaire ne sera réalisée à la prise en charge habituelle des patients, sinon la diffusion de musique chez les patients du groupe musicothérapie. Aucun risque n'est donc attendu lors de la mise en application de cette recherche.

Les informations recueillies seront traitées confidentiellement par informatique. A tout moment les patients pourront exercer leur droit d'accès aux informations, ainsi que leur droit de rectification, comme cela est prévu par la loi informatique et liberté »(article40).

Ce projet d'étude sera évalué par le Comité de Protection des Personnes de Saint Germain en Laye et déclaré à la Commission Nationale de l'Informatique et des Liberté (C.N.I.L.)

Une lettre d'information sera remise aux patients conscients mais sans communication verbale à l'inclusion. Ces patients seront informés oralement. Leur consentement de participation sera systématique recherché et attesté par la signature du formulaire de consentement de participation dédié. En cas d'incapacité physique de signer, une attention particulière sera apportée aux manifestations non verbales témoignant de la volonté du patient de ne pas participer à cette recherche.

Pour les patients non communicants et non conscients, une lettre d'information sera remise aux proches. Dans tous les cas, une lettre d'information sera remise aux patients après inclusion.

Aspects statistiques

Il sera procédé à une analyse unique. Les données seront analysées sur un mode univarié. Les variables qualitatives seront comparées par un test de Khi deux éventuellement corrigé selon la méthode de Yates selon la taille des effectifs ou par la méthode exacte de Fisher. Les variables quantitatives seront comparées par un test t ou test de Mann et Whitney selon la taille et la distribution des effectifs.

Un $p < 0,05$ sera considéré comme significatif.

Investigateurs

Stéphane LEGRIEL (1) (Investigateur coordonateur), Gwenaëlle JACQ (1) (Collaboratrice de l'investigateur), Matthieu RESCHE-RIGON (2) (Méthodologie et analyse statistique)

(1) Service de réanimation polyvalente,
Centre hospitalier de Versailles – Site André Mignot
177 rue de Versailles, 78157 LE CHESNAY CEDEX
tel : 01.39.63.88.39
fax : 01.39.63.86.88

(2) Département de Biostatistique et Informatique Médicale
CHU Saint Louis
1 avenue Claude Vellefaux
75010 PARIS
Tel : 01.42.49.97.42
Fax : 01.42.49.97.45

Conditions de publication

Les résultats de cette étude seront présentés à des congrès scientifiques et soumis pour publication dans une revue internationale à comité de lecture.

Bibliographie

1. Puntillo KA. Pain experiences of intensive care unit patients. Heart Lung 1990;19:526-33.
2. Fourrier F. Mieux vivre la réanimation. Réanimation 2010;19:191-203.
3. Van Rompaey B, Elseviers MM, Schuurmans MJ, Shortridge-Baggett LM, Truijen S, Bossaert L. Risk factors for delirium in intensive care patients: a prospective cohort study. Crit Care 2009;13:R77.
4. Boer KR, van Ruler O, van Emmerik AA, et al. Factors associated with posttraumatic stress symptoms in a prospective cohort of patients after abdominal sepsis: a nomogram. Intensive Care Med 2008;34:664-74.
5. Puntillo KA, Morris AB, Thompson CL, Stanik-Hutt J, White CA, Wild LR. Pain behaviors observed during six common procedures: results from Thunder Project II. Crit Care Med 2004;32:421-7.
6. Payen JF, Chanques G. [Pain management]. Ann Fr Anesth Reanim 2008;27:633-40.
7. Sauder P, Andreoletti M, Cambonie G, et al. Sédation-analgésie en réanimation (nouveau-né exclu). Annales Françaises d'Anesthésie et de Réanimation 2008;27:541-51.
8. Payen JF, Bosson JL, Chanques G, Mantz J, Labarere J. Pain assessment is associated with decreased duration of mechanical ventilation in the intensive care unit: a post Hoc analysis of the DOLOREA study. Anesthesiology 2009;111:1308-16.
9. Payen JF, Bru O, Bosson JL, et al. Assessing pain in critically ill sedated patients by using a behavioral pain scale. Crit Care Med 2001;29:2258-63.
10. Brook AD, Ahrens TS, Schaiff R, et al. Effect of a nursing-implemented sedation protocol on the duration of mechanical ventilation. Crit Care Med 1999;27:2609-15.
11. Kress JP, Pohlman AS, O'Connor MF, Hall JB. Daily interruption of sedative infusions in critically ill patients undergoing mechanical ventilation. N Engl J Med 2000;342:1471-7.
12. Tanios MA, de Wit M, Epstein SK, Devlin JW. Perceived barriers to the use of sedation protocols and daily sedation interruption: a multidisciplinary survey. J Crit Care 2009;24:66-73.
13. Mantz J. [Weaning from the ventilator modalities and consequences]. Ann Fr Anesth Reanim 2008;27:611-6.
14. Cepeda MS, Carr DB, Lau J, Alvarez H. Music for pain relief. Cochrane Database Syst Rev 2006:CD004843.
15. Nilsson S, Kokinsky E, Nilsson U, Sidenvall B, Enskar K. School-aged children's experiences of postoperative music medicine on pain, distress, and anxiety. Paediatr Anaesth 2009;19:1184-90.
16. Klassen JA, Liang Y, Tjosvold L, Klassen TP, Hartling L. Music for pain and anxiety in children undergoing medical procedures: a systematic review of randomized controlled trials. Ambul Pediatr 2008;8:117-28.
17. Whitehead-Pleaux AM, Zebrowski N, Baryza MJ, Sheridan RL. Exploring the effects of music therapy on pediatric pain: phase 1. J Music Ther 2007;44:217-41.
18. Avers L, Mathur A, Kamat D. Music therapy in pediatrics. Clin Pediatr (Phila) 2007;46:575-9.
19. Bradt J, Dileo C, Grocke D. Music interventions for mechanically ventilated patients. Cochrane Database Syst Rev 2011:CD006902.
20. Mahon EM, Mahon SM. Music therapy: a valuable adjunct in the oncology setting. Clin J Oncol Nurs 2011;15:353-6.

21. Mansky PJ, Wallerstedt DB. Complementary medicine in palliative care and cancer symptom management. Cancer J 2006;12:425-31.
22. Bausewein C, Booth S, Gysels M, Higginson I. Non-pharmacological interventions for breathlessness in advanced stages of malignant and non-malignant diseases. Cochrane Database Syst Rev 2008:CD005623.
23. Mossler K, Chen X, Heldal TO, Gold C. Music therapy for people with schizophrenia and schizophrenia-like disorders. Cochrane Database Syst Rev 2011:CD004025.
24. Bradt J, Dileo C, Grocke D, Magill L. Music interventions for improving psychological and physical outcomes in cancer patients. Cochrane Database Syst Rev 2011:CD006911.
25. Jaber S, Bahloul H, Guetin S, Chanques G, Sebbane M, Eledjam JJ. [Effects of music therapy in intensive care unit without sedation in weaning patients versus non-ventilated patients]. Ann Fr Anesth Reanim 2007;26:30-8.
26. Magill-Levreault L. Music therapy in pain and symptom management. J Palliat Care 1993;9:42-8.
27. Gerdner LA. Effects of individualized versus classical "relaxation" music on the frequency of agitation in elderly persons with Alzheimer's disease and related disorders. Int Psychogeriatr 2000;12:49-65.
28. Roy M, Peretz I, Rainville P. Emotional valence contributes to music-induced analgesia. Pain 2008;134:140-7.
