# Supplementary material for: Music for pain relief during bed bathing of mechanically ventilated patients: A pilot study
Source: PLoS One. 2018 Nov 14;13(11):e0207174. doi: 10.1371/journal.pone.0207174 (PMC6235356; doi:10.1371/journal.pone.0207174)
Supplement: S2 File — (PDF) [file pone.0207174.s002.pdf]

Evaluation of the analgesic effect of music in critically ill patients during potentially painful nursing procedures: a pilot study (Painkiller)

Investigators

Stéphane LEGRIEL, MD (1) (Coordinating investigator)

Gwenaëlle JACQ, RN(1) (Investigator collaborator)

Matthieu RESCHE-RIGON, MD, PhD (2) (Methodologist)

(1) Intensive Care Department, Centre Hospitalier de Versailles - Site André Mignot, 177 rue de Versailles, 78150 Le Chesnay cedex, France

Tel: +33 139 638 356 Fax: +33 139 638 688

(2) SBIM Biostatistics and Medical information, CHU Saint Louis, 1 avenue Claude Vellefaux, 75010 PARIS, FRANCE

Tel : +33 142.499 742 Fax : +33 142 499 745

Sponsor

French public funding agency *Délégation à la Recherche Clinique et à l'Innovation* (DRCI), Versailles, France.

SUMMARY

1. Summary of the research study 3
2. Scientific justification 4
3. Objectives and outcomes 6
   3.1. Primary objective 6
   3.2. Secondary objectives 6
   3.3. Primary outcome 6
   3.4. Secondary outcomes 7
4. Conception of the study 7
   4.1 Study design 7
   4.2 Sample size 7
   4.3 Eligibility criteria 7
      4.3.1 Inclusion criteria 7
   4.3.2 Exclusion criteria 8
5. Intervention 8
   5.1 Modalities of enrolment 8
   5.2 Evaluation 8
   5.3 Music intervention 8
   5.4 Common modalities in both groups 9
6. Data collection 9
7. Ethic and regulary considerations 10
8. Statistics 10
9. Rules relating to publication 10
10. References 11

1. Summary of the research study

Sponsor	French public funding agency *Délégation à la Recherche Clinique et à l'Innovation* (DRCI), Versailles, France
Investigators	Coordinating/Principal: Stéphane LEGRIEL, MD<br>Investigator collaborator : Gwenaëlle JACQ, RN
Title	Evaluation of the analgesic effect of music in critically ill patients during potentially painful nursing procedures: a pilot study (Painkiller)
Justification/ Context	Among critically ill patients who are able to communicate, nearly 60% report pain during their intensive care unit (ICU) stay. Pain's causes are numerous and essentially related to the care management and invasive procedures (catheters, drains removal, post-operative care, patient mobilization during bed bathing and nursing care procedures, wound care, tracheal suctioning...). Pain management relies on a simple strategy that associates: prevention, assessment, pharmacological and nonpharmacological pain treatment. However, whereas pain control in intensive care unit relies mainly on pharmacotherapy, non-pharmacological interventions such as music have been evaluated in various other settings such as pediatrics, oncology, neurology and psychiatry. In these patients, a recent Cochrane group metaanalysis concludes to a reduction in pain intensity and analgesic requirements. In critically ill patients undergoing mechanical ventilation, music effects have been evaluated in another Cochrane metaanalysis of studies demonstrating a decrease of anxiety occurrence, heart rate, respiratory rate but also blood pressure. Thus, it appears interesting to assess the therapeutic value of music as a nursing intervention in potentially painful nursing procedures in the intensive care unit.
Objectives	*Primary*: To evaluate the length of pain exposure for critically ill patients unable to communicate undergoing mechanical ventilation, whether or not receiving music, during nursing care.<br>*Secondary*: To evaluate the occurrence of peak pain exposure for critically ill patient unable to communicate undergoing mechanical ventilation, whether or not receiving music, during nursing care.
Study design	Prospective, interventional, comparative single center study
Inclusion criteria	Adults patients older than 18 years of age, admitted in intensive care unit, receiving invasive mechanical ventilation, and with a Richmond Agitation Sedative Scale (RASS) score between -3 and +4
Exclusion criteria	Patients receiving neuromuscular blocking agents or patients with a RASS score <-3
Outcomes	*Primary:* Proportion of pain exposure during nursing procedures.<br>Total length of pain exposure (minutes) to a BPS score over or equal to 5 divided by total length of nursing procedure<br>*Secondary:* Proportion of maximal pain exposure during nursing procedures Total length of exposure to the maximal BPS score (over or equal to 5) among total length of nursing procedure
Sample size	Pilot study, whose purpose is to obtain preliminary data and for which it is not possible to calculate a study sample size. The number of patients to be included was fixed at 60, ie 30 patients in each group (morning bed bathing and wound care with and without music).
Intervention	Eligible patients will be consecutively included. The first 30 patients will be included in the control group and the next 30 patients in the intervention group. In the intervention group, patients will be exposed to music during the morning bed bath, using headphones connected to an MP3 converter via a Bluetooth connection. The same selection of passages by Mozart will be used in all patients. The music will start at the same time as the bed bath and continued throughout the bath and for 30 minutes after the end of the bath. Sound intensity will do not exceed 60 decibels. In both groups, BPS scores will be determined by a nurse, who will not be involved in providing the bath. The BPS score will be determined just before the bath; at completion of the bath; and 30, 60, and 120minutes after the end of the bath. During the bath, the BPS score will be assessed in cases of variations of BPS score ≥5. Total length of the bath will be recorded.
Statistical analysis	Quantitative parameters will be described as median (interquartile range [IQR]) and qualitative parameters as number (%). All tests will be two-tailed at the 0.05 significance level with a two-sided hypothesis.

2. Scientific justification

Among critically ill patients who are able to communicate, nearly 60% report pain during their intensive care unit (ICU) stay.[1,2] Pain experience in the ICU has been demonstrated associated with the occurrence of delirium[3] and post-traumatic stress.[4] Pain's causes are numerous and essentially related to the care management and invasive procedures (catheters, drains removal, post-operative care, patient mobilization during bed bathing and nursing care procedures, wound care, tracheal suctioning...).[5,6] Pain management relies on a simple strategy that associates: prevention, assessment, pharmacological and nonpharmacological pain treatment. Recent guidelines strongly recommended further effort take pain into account during patients' management in the ICU.[7]

Pain assessment is remains a difficult problem and frequently underestimated in critically ill patients.[6] Use of pain assessment tools is mandatory because it allows adequate control of analgesia by nurse and physician staff.[8] Among the numerous available tools, patient self-reporting is the best indicator of pain level specifically when using pain rating scale (numeric pain scale-NPS or visual analog pain scale-VAS). In the non-communicative critically ill patients, the use of a behavior pain tool is also recommended and the behavioral pain scale (BPS) has been validated in the intensive care unit as reliable and reproductive.[9]

Whereas pain control in intensive care unit relies mainly on pharmacotherapy [6], several studies have recently shown that regular interruptions in the administration of sedative drugs was associated with a significant reduction in the duration of invasive mechanical ventilation use and a decrease of delirium occurrence, but also in lower length of ICU a stay.[10, 11] In this strategy, pain assessment should be probably reinforced in order to do not expose patients to further pain-related complications. Indeed, other studies have reported higher short-term[12] but also long-term[13] morbidity when patients experience pain in the ICU, namely: patient-

ventilator dyssynchrony, agitation, tachycardia, hypertension, unplanned extubation, and post-traumatic syndrome disorder. [12, 13]

Thus, it is important to propose complementary and alternative therapies for pain management. Non-pharmacological interventions such as music have been evaluated in many studies.[14] A growing body of literature examines the opportunity to deliver a music intervention such as in pediatrics [15-18], oncology [19-21] or neurology and psychiatry [22-24]. An assessment of the value of music therapy on pain has been the subject of a Cochrane group metaanalysis which concludes to a reduction in pain intensity and analgesic requirements. In critically ill patients undergoing mechanical ventilation, music effects have been evaluated in another Cochrane metaanalysis of studies demonstrating a decrease of anxiety occurrence, heart rate, respiratory rate but also blood pressure.[19, 25]

This approach is particularly interesting since its physiological mechanisms are now better elucidated. Music effects have thus been demonstrated on the different components of pain:

- sensory component by exerting an inhibitory effect on the afferent fibers: activation of the pathways of pain and brain transmission with modulation and control the transmission of the nociception signals at different stages of the nervous system[26]
- cognitive component by distracting attention from memories or images : psychic representation of pain in relation to past painful experiences and events associated with painful episodes[27]
- affective component by changing the state of mood and by stimulating the release of endorphins: gives pain a character more or less painful, unpleasant, bearable according to individuals, may even evolve towards states of anxiety or depression[28]

- -behavioral component by acting on hypertonia and psychomotricity: behavior individual in case of pain

Thus, at a time when expert recommendations encourage the development of assessment tools, behaviors and care giving organizations that would improve patient's wellbeing in the ICU, it appears interesting to assess the therapeutic value of music as a nursing intervention in potentially painful nursing procedures in the intensive care unit.

3. Objectives and outcomes

3.1. Primary objective

To evaluate the length of pain exposure for critically ill patients unable to communicate undergoing mechanical ventilation, whether or not receiving music, during nursing care.

3.2. Secondary objectives

To evaluate the occurrence of peak pain exposure for critically ill patient unable to communicate undergoing mechanical ventilation, whether or not receiving music, during nursing care.

3.3. Primary outcome

Total length of pain exposure (minutes) to a BPS score over or equal to 5 divided by total length of nursing procedure

3.4. Secondary outcomes

Total length of exposure to the maximal BPS score (over or equal to 5) among total length of nursing procedure

4. Conception of the study

4.1 Study design

A prospective, interventional, comparative, single center study.

4.2 Sample size

This is a pilot study, whose purpose is to obtain preliminary data and for which it is not possible to calculate a study sample size. The number of patients to be included was fixed at 60, ie 30 patients in each group (morning bed bathing and wound care with and without music).

4.3 Eligibility criteria

4.3.1 Inclusion criteria

-adults patients admitted in intensive care unit if they were older than 18 years of age

-receiving invasive mechanical ventilation,

-with a Richmond Agitation Sedative Scale (RASS) score between -3 and +4.

4.3.2 Exclusion criteria

-adults patients receiving neuromuscular blocking agents

-adults patients with a RASS score <-3

5. Intervention

5.1 Modalities of enrolment

In this study, eligible patients will be consecutively included. The first 30 patients will be included in the control group and the next 30 patients in the intervention group.

5.2 Evaluation

As bed bathing is performed in all patients and in a standardized manner, we will evaluate the potential effects of music during the morning bed bath. Evaluation may also be performed during bedside surgical dressing.

5.3 Music intervention

In the intervention group, patients will be exposed to music during the morning bed bath, using headphones connected to an MP3 converter via a Bluetooth connection. The same selection of passages by Mozart will be used in all patients. The music will start at the same time as the bed bath and continued throughout the bath and for 30 minutes after the end of the batch. Sound intensity will do not exceed 60 decibels.

5.4 Common modalities in both groups

BPS scores will be determined by a nurse, who will not be involved in providing the bath. The BPS score will be determined just before the bath; at completion of the bath; and 30, 60, and 120minutes after the end of the bath. During the bath, the BPS score will be assessed in cases of variations of BPS score ≥5. Total length of the bath will be recorded. All patients will receive analgesia and sedation as needed, according to the standard protocol in our unit, which involves various titrated combinations of midazolam, propofol, morphine, and/or sufentanil.

6. Data collection

Demographic and clinical data will be collected prospectively at the bedside on a standardized form. Demographics data: age, gender, reason for ICU admission (medical, emergency surgery, scheduled surgery), comorbidities, reason for mechanical ventilation, ICU length of stay before inclusion. Patients characteristics at inclusion: presence of tracheotomy, chest tubes, central venous catheter, arterial line, gastric tube, surgical drain tube. Nursing care characteristics will be assessed in both groups at baseline; during; and 30, 60, and 120 minutes after the end of the evaluated nursing care. Total length of nursing procedure will be recorded. Data related to pain assessment: Total length of pain exposure (minutes) to a BPS score over or equal to 5; and total length of exposure to the maximal BPS score (over or equal to 5).

7. Ethic and regulary considerations

Our local ethics committee approved this prospective pilot study (*Comité de Protection des Personnes, Paris – Ile de France VI*, 5 October 2012, #12044). Written informed consent from a relative or surrogate will be obtained for each participant before study inclusion and, whenever possible, from the patients before ICU discharge. This study will be performed in compliance with the ethical standards of the institutional and national research committees and with the 1964 Declaration of Helsinki and its later amendments.

8. Statistics

Quantitative parameters will be described as median (interquartile range [IQR]) and qualitative parameters as number (%). Categorical variables will be compared using Fisher's exact test and continuous variables using the t test or Mann Whitney or Wilcoxon tests depending to sampling distribution. All tests will be two-tailed at the 0.05 significance level with a two-sided hypothesis.

9. Rules relating to publication

Publication of the main results will be presented a scientific congress and submitted for publication in a peer review international journal.

10. References

1 Puntillo KA. Pain experiences of intensive care unit patients. Heart Lung 1990;19:526-33.

2. Fourrier F. Mieux vivre la réanimation. Réanimation 2010;19:191-203.

3. Van Rompaey B, Elseviers MM, Schuurmans MJ, Shortridge-Baggett LM, Truijen S, Bossaert L. Risk factors for delirium in intensive care patients: a prospective cohort study. Crit Care 2009;13:R77.

4. Boer KR, van Ruler O, van Emmerik AA, et al. Factors associated with posttraumatic stress symptoms in a prospective cohort of patients after abdominal sepsis: a nomogram. Intensive Care Med 2008;34:664-74.

5. Puntillo KA, Morris AB, Thompson CL, Stanik-Hutt J, White CA, Wild LR. Pain behaviors observed during six common procedures: results from Thunder Project II. Crit Care Med 2004;32:421-7.

6. Payen JF, Chanques G. [Pain management]. Ann Fr Anesth Reanim 2008;27:633-40.

7. Sauder P, Andreoletti M, Cambonie G, et al. Sédation-analgésie en réanimation (nouveau-né exclu). Annales Françaises d'Anesthésie et de Réanimation 2008;27:541-51.

8. Payen JF, Bosson JL, Chanques G, Mantz J, Labarere J. Pain assessment is associated with decreased duration of mechanical ventilation in the intensive care unit: a post Hoc analysis of the DOLOREA study. Anesthesiology 2009;111:1308-16.

9. Payen JF, Bru O, Bosson JL, et al. Assessing pain in critically ill sedated patients by using a behavioral pain scale. Crit Care Med 2001;29:2258-63.

10. Brook AD, Ahrens TS, Schaiff R, et al. Effect of a nursing-implemented sedation protocol on the duration of mechanical ventilation. Crit Care Med 1999;27:2609-15.

11. Kress JP, Pohlman AS, O'Connor MF, Hall JB. Daily interruption of sedative infusions in critically ill patients undergoing mechanical ventilation. N Engl J Med 2000;342:1471-7.

12. Tanios MA, de Wit M, Epstein SK, Devlin JW. Perceived barriers to the use of sedation protocols and daily sedation interruption: a multidisciplinary survey. J Crit Care 2009;24:66-73.

13. Mantz J. [Weaning from the ventilator modalities and consequences]. Ann Fr Anesth Reanim 2008;27:611-6.

14. Cepeda MS, Carr DB, Lau J, Alvarez H. Music for pain relief. Cochrane Database Syst Rev 2006:CD004843.

15. Nilsson S, Kokinsky E, Nilsson U, Sidenvall B, Enskar K. School-aged children's experiences of postoperative music medicine on pain, distress, and anxiety. Paediatr Anaesth 2009;19:1184-90.

16. Klassen JA, Liang Y, Tjosvold L, Klassen TP, Hartling L. Music for pain and anxiety in children undergoing medical procedures: a systematic review of randomized controlled trials. Ambul Pediatr 2008;8:117-28.

17. Whitehead-Pleaux AM, Zebrowski N, Baryza MJ, Sheridan RL. Exploring the effects of music therapy on pediatric pain: phase 1. J Music Ther 2007;44:217-41.

18. Avers L, Mathur A, Kamat D. Music therapy in pediatrics. Clin Pediatr (Phila) 2007;46:575-9.

19. Bradt J, Dileo C, Grocke D. Music interventions for mechanically ventilated patients. Cochrane Database Syst Rev 2011:CD006902.

20. Mahon EM, Mahon SM. Music therapy: a valuable adjunct in the oncology setting. Clin J Oncol Nurs 2011;15:353-6.

21. Mansky PJ, Wallerstedt DB. Complementary medicine in palliative care and cancer symptom management. Cancer J 2006;12:425-31.

22. Bausewein C, Booth S, Gysels M, Higginson I. Non-pharmacological interventions for breathlessness in advanced stages of malignant and non-malignant diseases. Cochrane Database Syst Rev 2008:CD005623.
23. Mossler K, Chen X, Heldal TO, Gold C. Music therapy for people with schizophrenia and schizophrenia-like disorders. Cochrane Database Syst Rev 2011:CD004025.
24. Bradt J, Dileo C, Grocke D, Magill L. Music interventions for improving psychological and physical outcomes in cancer patients. Cochrane Database Syst Rev 2011:CD006911.
25. Jaber S, Bahloul H, Guetin S, Chanques G, Sebbane M, Eledjam JJ. [Effects of music therapy in intensive care unit without sedation in weaning patients versus non-ventilated patients]. Ann Fr Anesth Reanim 2007;26:30-8.
26. Magill-Levreault L. Music therapy in pain and symptom management. J Palliat Care 1993;9:42-8.
27. Gerdner LA. Effects of individualized versus classical "relaxation" music on the frequency of agitation in elderly persons with Alzheimer's disease and related disorders. Int Psychogeriatr 2000;12:49-65.
28. Roy M, Peretz I, Rainville P. Emotional valence contributes to music-induced analgesia. Pain 2008;134:140-7.
